# Explicit Versus Implicit “Halal” Information: Influence of the Halal Label and the Country-of-Origin Information on Product Perceptions in Indonesia

**DOI:** 10.3389/fpsyg.2018.00382

**Published:** 2018-03-22

**Authors:** Dominika Maison, Marta Marchlewska, Dewi Syarifah, Rizqy A. Zein, Herison P. Purba

**Affiliations:** ^1^Faculty of Psychology, University of Warsaw, Warsaw, Poland; ^2^Institute of Psychology, Polish Academy of Sciences, Warsaw, Poland; ^3^Department of Industrial and Organizational Psychology, Airlangga University, Surabaya, Indonesia; ^4^Department of Personality and Social Psychology, Airlangga University, Surabaya, Indonesia

**Keywords:** halal certification, country-of-origin, explicit versus implicit product information, religion-based purchase behavior, Muslim community

## Abstract

Halal refers to what is permissible in traditional Islamic law. Food that meets halal requirements is marked by a halal label on the packaging and should be especially attractive to those Muslims who follow the set of dietary laws outlined in the Quran. This research examines the role of the halal label (explicit cue) and the country-of-origin (COO) (implicit cue) in predicting positive product perceptions among Muslim consumers. We hypothesized that when an explicit sign of “halalness” (i.e., halal label) relating to a particular product is accompanied by an implicit sign of anti*-*“halalness” (i.e., non-Islamic COO information), Muslim consumers who pay attention to the dietary laws of Islam would have negative perceptions of such a product. We tested our assumptions in an experiment conducted among Indonesian participants who declared themselves as Muslims (*n* = 444). We manipulated: (a) exposure to the halal label, and (b) the COO information. Religion-based purchase behavior was measured as a moderator variable. Positive product perceptions were measured as a dependent variable. The results showed that the halal label itself had limited influence on product perceptions. However, we found that positive product perceptions significantly decreased among people who were high in religion-based purchase behavior in response to exposure to non-Islamic COO information accompanied by a halal label. In conclusion, people who are high (vs. low) in religion-based purchase behavior do not seem to trust halal-labeled food produced in a country with other than an Islamic tradition.

## Introduction

Consumers rely on different cues when making their purchase decisions. Some of these cues are directly related to the product quality (i.e., *intrinsic quality attributes*; Blanco et al., 2007). They imbue the product with its functionality and cannot be altered without changing the nature of the product itself (Blanco et al., 2007). For example, when taking into account food purchases, one person may search for products that taste or smell in a particular way (e.g., sweet) or which are made with healthy ingredients. Another person, however, may focus on the extrinsic features of a particular product (i.e., *extrinsic quality attributes;* Blanco et al., 2007) that are related to the product but are not physically a part of it, such as the price, brand, region of origin, or packaging characteristics (Verlegh and Steenkamp, 1999; Watson and Wright, 2000; Blanco et al., 2007; Maison and Gregg, 2016). In this research, we seek to examine whether extrinsic cues related to the products, namely: (a) the halal label, and (b) the country-of-origin (COO), may influence positive product perceptions among Muslim consumers who pay attention to the dietary laws of Islam.

Halal means “permissible” in Arabic and refers to the nature, origin, and processing methods of the food (Abdul et al., 2009). The halal label is important for Muslims because it serves as an explicit symbol of religious permission that is assigned to products which have been certified as meeting Islamic Food Laws (Ahmed, 2008; Abdul et al., 2009; Wilson and Liu, 2010). For this reason, in many countries (e.g., Malaysia, Turkey but also the United Kingdom) food prepared according to Muslim dietary restrictions is certified by a halal label on the packaging. In the case of very religious Muslims, the halal label is a key feature of any food that they eat. In line with this logic, Verbeke et al. (2013) showed that Muslim consumers in Belgium who scored high (vs. low) in the importance attached to a certified halal label were willing to pay a higher price for certified halal (vs. not) labeled meat at an Islamic butcher’s shop. The results of other studies conducted in Islam-majority countries (i.e., Malaysia or Indonesia) have demonstrated that Muslim consumers declare that halal certification is important to them in making their purchase decisions (Amat et al., 2014; Sukesti and Budiman, 2014; Farhan and Andriansyah, 2016).

On the other hand, more and more researchers point to the fact that there is an uncertainty regarding halal quality among Muslim consumers (Bonne and Verbeke, 2007; Lever and Miele, 2012). In other words, the levels of Muslim trust in the health and safety of halal certified products may vary depending on certain conditions (Shafiq et al., 2015). For example, Shafiq and colleagues (2015) demonstrated that participants only trusted the green or black and white halal logos and evaluated others as fake, though the halal logo can be officially modified as desired by the manufacturer and can take different colors (Mahmood, 2011). This may mean that in some situations the halal label can even detract Muslim consumers from perceiving the products in a positive manner. In our research, we assumed that this might be the case when an explicit sign of “halalness” (i.e., halal label) is accompanied by an implicit (or indirect) sign of anti-“halalness” (i.e., non-Islamic COO).

The halal label placed on packaging gives explicit information about the “halalness” of the product. However, this information can also be provided in a more implicit way, for example by: (a) using an Arabic name for the product; (b) using Arabic letters on the packaging; or (c) placing a label indicating that the product was manufactured in a country where Islam is the dominant religion (Verlegh and Steenkamp, 1999; Abdul et al., 2009). In our study, we focus on the last of the aforementioned factors. Research on the influence of COO information on product perception shows that it can have both a positive or negative character (Hong and Wyer, 1989; Obermiller and Spangenberg, 1989; Liefeld, 1993; Elliott and Cameron, 1994; Maheswaran, 1994; Verlegh and Steenkamp, 1999; Hamin and Elliott, 2006; Bolliger and Réviron, 2008; Koschate-Fischer et al., 2012; Rios et al., 2014). According to the “made-in” phenomenon, COO information may result in positive (vs. negative) product perceptions when the consumer believes (vs. does not believe) that the country which produced the particular product specializes in manufacturing these types of goods (Verlegh and Steenkamp, 1999). As halal products are strictly related to Islam, one may expect Muslims to doubt the “halalness” of a product which was produced in a non-Islamic country (e.g., Muslim-minority country). In this way, the COO information can serve as an implicit symbol of “halalness” (vs. “haramness” – which is an Arabic term meaning sinful, forbidden products).

### Overview of the Current Research

Information regarding whether a particular product is permissible (vs. forbidden) for Muslims can be provided in different ways. One of them is by putting a halal logo on the product packaging, which is a well-known practice in many Islamic countries. Another packaging feature that may serve as a sign of permission or prohibition for Muslims is the COO information (Leclerc et al., 1994; Gürhan-Canli and Maheswaran, 2000; Watson and Wright, 2000). A number of studies showed a general bias against foreign (in favor of domestic) products, which is manifest in negative product perceptions and reduced buying intentions (Özsomer and Cavusgil, 1991; Peterson and Jolibert, 1995; Verlegh and Steenkamp, 1999). If this refers not only to national but also religious affiliation, Muslims should prefer those goods which are produced in a country where Islam is the dominant religion. On the other hand, they should reject those products whose COO is not strongly related to Islam. Moreover, it is possible that Islamic (vs. non-Islamic) countries are perceived by Muslims as having more (vs. less) expertise in the halal manufacturing industry and, as a consequence, are more (vs. less) trusted as being able to follow halal rules properly. Thus, those Muslims for whom religion plays a major role in their consumer choices may feel confused by halal-labeled products which were manufactured in countries dominated by religions other than Islam (e.g., Christianity).

The main goal of our research was to explore the influence of the COO and halal information on positive product perceptions. Prior studies regarding the relationship between “halalness” and positive product perceptions were mainly correlational. In this study, we decided to go beyond simple declarations and used an experimental design to test our assumptions.

First, we aimed to test the influence of COO information on positive product perceptions. We assumed that a non-Islamic COO would negatively influence product perceptions and, thus, we expected significant positive effects of: (H1) Islamic COO information (vs. non-Islamic COO information), and (H2) no information on COO (vs. non-Islamic COO information) on positive product perceptions. We expected these effects to be especially pronounced among individuals who focus on religious principles when making food choices, thus among people who are high (vs. low) in religion-based purchase behavior (H3).

Second, we explored the influence of the halal label on positive product perceptions. We expected the halal label displayed (vs. not displayed) on the packaging to increase positive product perceptions (H4). We assumed that this effect would be especially pronounced among people who are high (vs. low) in religion-based purchase behavior (H5).

Finally, we aimed to test if people who are high (vs. low) in religion-based purchase behavior would react negatively to the inconsistency between COO information and halal labeling. Specifically, we assumed that religion-based purchase behavior would negatively predict product perceptions in the non-Islamic COO information (vs. Islamic or no COO information) condition accompanied by a halal label (vs. not accompanied) (H6).

## Materials and Methods

### Participants and Procedure

The study was conducted among 509 Indonesian internet users. Participants were asked if they would like to participate in consumer research collecting opinions about a new product (cake) entering the Indonesian market. Those who agreed, were automatically redirected to the survey. Ethical approval was granted by the Research Ethics Committee of the Institute for Social Studies (University of Warsaw). Because we expected effects only for Muslim participants, data from those who identified themselves as non-Muslims were excluded from our analyses (*n* = 65). Thus, the final sample included 444 Muslim participants: 343 women, 101 men, aged 17–59 (*M_age_* = 22.18, *SD* = 5.47). Participation was voluntary and anonymous, with no remuneration offered to the participants. We used IBM Spss (2016) and PROCESS (Hayes, 2018) to conduct our analyses.

First, the participants were asked to fill out a measure of religion-based purchase behavior. Next, they were randomly assigned to one of the experimental conditions. The study design employed a 2 (halal label vs. no halal label) × 3 (non-Islamic COO = England vs. Islamic COO = Turkey vs. no information about COO) manipulation. All of the participants were asked to look at the screen photograph of the FMCG food product (i.e., cake; see **Supplementary Figure S1**). Cake was chosen as an example of a neutral product not violating halal rules (i.e., low risk of containing items forbidden to Muslims, e.g., alcohol or pork). Depending on the condition (halal label manipulation), the participants were exposed to a product with the halal logo placed on the packaging (*n =* 229) or not (*n =* 215). The size, form, and color of the halal label was similar to those used in Indonesia. COO information was placed above the picture of the product. Depending on the condition (COO manipulation), the participants were informed that the COO of the product was England (non-Islamic country condition; *n =* 135) or that the COO of the product was Turkey (Islamic country condition; *n =* 155) or they received no information on the COO (no COO information condition; *n =* 154). Afterward, the participants answered a set of questions about their perception of the product.

### Measures

#### Religion-Based Purchase Behavior

It was measured with four items (1) “ I always take a careful look at the origin of everything I consume because of my religion.”, (2) “Due to religious issues, I prefer to buy a halal food product which has a halal label displayed on the product at the point of purchase.”, (3) “My religion is the main reason that I choose a halal food product wherever I go to get a meal.”, (4) “A halal food product fits harmoniously with my religious values.”. The participants were asked to rate each statement on a scale from 1 = *completely disagree* to 5 = *completely agree*, α = 0.89, *M =* 4.31, *SD =* 0.74. A higher mean score indicated higher religion-based purchase behavior.

#### Positive Product Perceptions

Positive product perceptions were measured with three items describing two sides of a continuum: (1) “This product does not look tasty at all” vs. “This product looks very tasty”; (2) “This product is definitely not good for me” vs. “This product is definitely good for me”; (3) “I definitely do not want to try this product” vs. “I definitely want to try this product.” The participants were asked to respond to these items using a scale from 1 (greater agreement with negative product perception items) to 4 (greater agreement with positive product perception items), α = 0.64, *M =* 2.89, *SD =* 0.39. A higher mean score indicated higher positive product perceptions.

## Results

First, we computed the correlation between religion-based purchase behavior and positive product perceptions across the conditions. This correlation was non-significant, *r*(442) = 0.05, *p* = 0.35. Second, the independent *t*-test revealed that participants in the halal label condition did not show significantly higher positive product perceptions (*M* = 2.90, *SD* = 0.37) than participants in the no halal label condition (*M* = 2.87, *SD* = 0.42), *t*(442) = 0.89, *p* = 0.37. Finally, we performed a hierarchical multiple regression analysis to investigate the effects of the variables coding experimental manipulations in interactions with religion-based purchase behavior on positive product perceptions. Religion-based purchase behavior was mean-centered prior to analyses. The halal label manipulation was coded as 0 = non-halal label condition, and 1 = halal label condition. The COO experimental conditions (non-Islamic COO vs. Islamic COO vs. no information about COO) were recoded into two dummy variables. The first dummy variable encoded the difference between an Islamic COO and a non-Islamic COO; the second dummy variable encoded the difference between no information about the COO and a non-Islamic COO. We additionally controlled for the following demographics: age and gender; coded 1 = woman; 0 = man (**Table 1**).

**Table 1:** Predictors of positive product perceptions.

	Step 1		Step 2		Step 3	
Variables	*B(SE)*	*p*	*B(SE)*	*p*	*B(SE)*	*p*
Age	-0.003 (0.003)	0.39	-0.003 (0.003)	0.40	-0.004 (003)	0.23
Gender (1 = women; 0 = men)	-0.05 (0.05)	0.31	-0.05 (0.05)	0.29	-0.06 (0.05)	0.16
Religion-based purchase behavior	0.03 (0.03)	0.31	-0.10 (0.05)	0.05	-0.01 (0.05)	0.91
Halal (vs. no Halal)	0.03 (0.04)	0.49	0.12 (0.07)	0.08	0.12 (0.07)	0.08
Islamic COO (vs. non-Islamic COO)	0.13 (0.05)	0.01	0.23 (0.07)	<0.001	0.23 (0.06)	<0.001
No COO (vs. non-Islamic COO)	0.08 (0.05)	0.09	0.10 (0.06)	0.09	0.10 (0.06)	0.11
Islamic COO × Halal			-0.21 (0.09)	0.02	-0.21 (0.09)	0.02
No COO × Halal			-0.05 (0.09)	0.60	-0.04 (0.09)	0.67
Halal × Religion-based purchase behavior			0.03 (0.05)	0.53	-0.21 (0.09)	0.02
Islamic COO × Religion-based purchase behavior			0.15 (0.06)	0.02	0.05 (0.09)	0.59
No COO × Religion-based purchase behavior			0.16 (0.06)	0.01	-0.02 (0.08)	0.82
Islamic COO × Religion-based purchase behavior × Halal					0.27 (0.13)	0.03
No COO × Religion-based purchase behavior × Halal					0.43 (0.12)	0.001
*F*	1.81	0.09	2.42	0.01	3.03	<0.001
*R*^2^	0.02		0.06		0.08	

In the first step, we investigated the effects of religion-based purchase behavior, halal (vs. non-halal) exposure, and two dummy variables. We found a significant positive effect of the Islamic COO condition, indicating that positive product perceptions were higher in the Islamic COO condition relative to the non-Islamic COO condition. Moreover, we found a marginally significant effect of the no information on the COO condition, indicating that positive product perceptions were higher when participants did not receive information on COO relative to the non-Islamic condition. We did not find any significant main effects of the halal (vs. non-halal) label condition or religion-based purchase behavior.

In the second step, we introduced five two-way interactions between all the predictors. We found a significant two-way interaction between the Islamic (vs. a non-Islamic) COO condition and exposure to the halal label condition. Simple slope analyses indicated that the effect of the Islamic COO (vs. non-Islamic COO) condition was positive albeit non-significant in the halal label condition, *B* = 0.02, *SE* = 0.07, *p* = 0.78, and positive and significant in the non-halal label condition, *B* = 0.22, *SE* = 0.06, *p* = 0.004, which suggested that the Islamic COO (vs. a non-Islamic COO) condition influenced positive product perceptions, especially when participants were not exposed to the halal label. We did not find significant two-way interactions between: (1) the no information on the COO (vs. non-Islamic COO) condition, and exposure (vs. no exposure) to the halal label; or (2) religion-based purchase behavior and exposure (vs. no exposure) to the halal label. However, we found significant two-way interactions between: (1) religion-based purchase behavior and the Islamic COO (vs. non-Islamic COO) condition, and (2) religion-based purchase behavior and the no information on the COO (vs. non-Islamic COO) condition. Simple slope analysis indicated that the effect of religion-based purchase behavior was negative and significant in the non-Islamic COO condition, *B* = -0.10, *SE* = 0.05, *p* = 0.04, and positive albeit insignificant in the Islamic condition, *B* = 0.05, *SE* = 0.05, *p* = 0.30 and in the no information on the COO condition, *B* = 0.07, *SE* = 0.05, *p* = 0.17.

**FIGURE 1:**
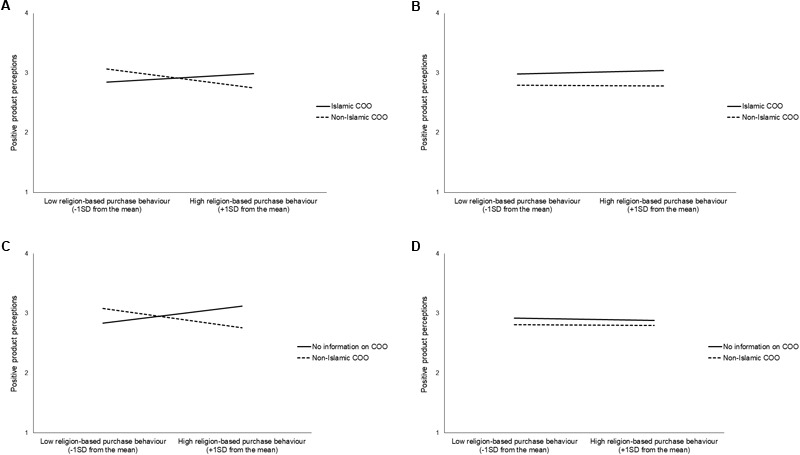
**(A)** Interaction effect of the Islamic (vs. non-Islamic) COO condition and religion-based purchase behavior on positive product perceptions among those in the halal condition. **(B)** Interaction effect of the Islamic (vs. non-Islamic) COO condition and religion-based purchase behavior on positive product perceptions among those in the non-halal condition. **(C)** Interaction effect of no information on the COO (vs. non-Islamic COO) condition and religion-based purchase behavior on positive product perceptions among those in the halal condition. **(D)** Interaction effect of no information on the COO (vs. non-Islamic COO) condition and religion-based purchase behavior on positive product perceptions among those in the non-halal condition.

In the third step, we introduced two three-way interactions between: (1) religion-based purchase behavior, the Islamic (vs. non-Islamic) COO condition, and the halal condition; and (2) religion-based purchase behavior, the no information on the COO (vs. non-Islamic COO) condition and the halal condition. Both interactions were significant. Further analyses revealed that the interaction effect of religion-based purchase behavior and an Islamic (vs. a non-Islamic) COO was significant only for the halal condition, *B* = 0.32, *SE* = 0.09, *p* < 0.001 (**Figure 1A**), and non-significant for the non-halal condition, *B* = 0.05, *SE* = 0.08, *p* = 0.58 (**Figure 1B**).

Similarly, the interaction effect of religion-based purchase behavior and no information on the COO (vs. non-Islamic COO) condition was significant only for the halal condition, *B* = 0.41, *SE* = 0.09, *p* < 0.001 (**Figure 1C**), and non-significant for the non-halal condition, *B* = -0.02, *SE* = 0.08, *p* = 0.83 (**Figure 1D**).

Simple slope analysis indicated that the effect of religion-based purchase behavior on positive product perception in the halal condition was negative and significant in the non-Islamic COO condition, *B* = -0.22, *SE* = 0.07, *p* = 0.002; positive and marginally significant in the Islamic COO condition, *B* = 0.10, *SE* = 0.06, *p* = 0.09; and positive and significant in the no information on COO condition, *B* = 0.19, *SE* = 0.06, *p* = 0.001.

## Discussion

In this paper, we examined the factors that influence positive product perceptions among Muslim consumers in Indonesia. We focused on two extrinsic cues related to food products: (a) the halal label, and (b) the COO, and a dispositional trait of the consumer, namely, religion-based purchase behavior. The results of our study confirmed that “halalness” is an important factor underlying product perceptions. We found, however, that both the effects of the halal label (explicit “halalness”) and the COO (implicit “halalness”) on product perceptions are complex and depend on many factors (e.g., whether an individual pays attention to the dietary laws of Islam and whether there is a consistency between implicit and explicit halal cues).

First of all, our study showed that exposure to Turkey – an Islamic country (vs. England – a non-Islamic country) as the COO strengthened positive product perceptions, indicating that Muslims prefer food produced in Islamic (vs. non-Islamic) countries. We also found a significant positive effect of no COO information (vs. England as the COO) on positive product perceptions. Thus, it seems that Muslim consumers have negative perceptions of food produced in non-Islamic countries and trust them even less than products that have been produced in an unknown country which provides confirmation for hypotheses H1 and H2. This is in line with studies showing a general bias against foreign products (Özsomer and Cavusgil, 1991; Peterson and Jolibert, 1995; Verlegh and Steenkamp, 1999) and may suggest that Muslim consumers are especially reluctant to buy food produced in non-Islamic countries. This effect, however, was only marginally significant and, therefore, should be treated with caution.

Second, we did not find any significant main effect of the halal label on positive product perceptions (hypotheses H4, H5 were rejected), however, we did find that the halal label significantly decreased the positive perceptions of the product when the product was introduced as coming from England (i.e., non-Islamic country). We did not observe this effect in the case of a product with Turkey as the COO (i.e., an Islamic country).

Finally, we found that positive product perceptions significantly decreased in response to exposure to non-Islamic COO information (i.e., a product manufactured in England vs. Turkey or no COO information) among people high (vs. low) in religion-based purchase behavior (confirmation of H6). Further analysis indicated that this effect was present only when non-Islamic COO information was accompanied by the halal label. Thus, it seems that those Muslims who follow the set of dietary laws outlined in the Quran may feel confused when facing a halal label accompanied by non-Islamic COO information and, as a result, reject this type of product. These results can be interpreted in terms of a cognitive dissonance (Festinger, 1957, 1964) that might have been evoked by receiving inconsistent information on the explicit “halalness” versus implicit anti-“halalness” of a particular product. Such a situation probably produces discomfort, which may be reduced by a definite rejection of the product that aroused the negative feelings. At the same time, it is also possible that the use of halal certification by non-Islamic countries may result in the product’s credibility being doubted by people who are high in religion-based consumer behavior, thus, being interpreted rather as a “cheap marketing trick” instead of reliable information.

Our findings corroborate and extend previous research on the role of “halalness” in shaping consumer choices. Although several previous studies evaluated the role of the halal label in shaping consumer decisions, they were mainly correlational and focused instead on the participants’ general opinions regarding halal certification rather than on their psychological responses to the products that carry a halal label. Previous studies found that Muslims differ with respect to trust in their health and the safety perceptions of halal food (Bonne and Verbeke, 2007; Lever and Miele, 2012; Shafiq et al., 2015). As demonstrated in our study, the participants who paid attention to the dietary laws of Islam did not trust in food that carried a halal label but was produced in a country where Islam is not a dominant religion (i.e., England). Thus, it seems that the influence of the halal label on positive product perceptions is more complex and also depends on other extrinsic features of a particular product (e.g., COO) as well as on individual consumer characteristics (e.g., religion-based purchase behavior).

Future research would do well to replicate these results in other countries and cultural and marketing contexts. Our study was conducted among Muslim participants living in Indonesia. The question is whether we may expect a similar pattern of results in the case of research conducted among: (a) Muslim consumers living in other Islamic countries (e.g., Malaysia), and (b) Muslim consumers living in non-Islamic countries (e.g., England). In fact, it is possible that Muslims who live in England would be more likely to trust British halal food than those Muslims who live in countries where Islam is the dominant religion (e.g., Indonesia). This relationship, however, may be moderated by different variables, such as the levels of assimilation or identification with national and religious in-groups. On the other hand, potentially fertile ground for future research would also be to investigate the reactions to “halalness” among non-Muslim consumers living in Islamic countries. This kind of research can be carried out, for example, in Indonesia, which is a place known not only for attaching great importance to halal certificates but also for its multiculturalism.

Furthermore, in our research we used only one type of product and exposed participants to a rather neutral type of food (i.e., cake), which did not contain any meat or alcohol and, thus, may be perceived by Muslims as less “risky” when it comes to breaking halal rules. Therefore, future research should elucidate more precisely the effects of a halal label and the COO information on consumers’ perceptions of different types of food – also those that may pose a threat to halal rules (e.g., animal products). Presumably, in the case of products with a high risk of breaking halal rules, the results may be even more robust than those obtained in our study as Muslim consumers should be more focused on detecting fake halal certificates.

Future research is also needed to understand the influence of the halal label on consumer choices in a real shopping setting with real products that can be carefully inspected by the consumer before making a purchase decision. For example, it would be interesting to find out which sign of “halalness” (implicit vs. explicit) attracts Muslim consumers’ attention first and how often they take additional actions (e.g., turning around the product in their hand and reading food labels) to find out whether a particular product meets the dietary laws of Islam. Also, when it comes to methodological improvements, in this research the moderator variable (i.e., religion-based purchase behavior) was measured at the beginning of the study which may unintentionally direct respondents’ attention to religious issues. Thus, future research should examine whether the pattern of results would be similar when measuring this variable at the end of the study.

Overall, the current results helped us understand the role of the halal label and the (Islamic vs. non-Islamic) COO information in shaping the choices of Muslim consumers who follow the set of dietary laws outlined in the Quran. Taking into account that adherents of Islam constitute the world’s second largest religious group (Desilver and Masci, 2017), there is no doubt that investigating purchasing decision factors among Muslim consumers is crucial. This type of knowledge can seep into practical use in a great variety of ways and should be especially valuable to all foreign companies entering the Islamic market.

## Ethics Statement

This study was carried out in accordance with the recommendations of Research Ethics Committee of the Institute for Social Studies (University of Warsaw) with informed consent from all subjects (online study). All subjects gave informed consent in accordance with the Declaration of Helsinki. The protocol was approved by the Research Ethics Committee of the Institute for Social Studies (University of Warsaw).

## Author Contributions

DM, DS, RZ, and HP contributed to the conception and design of the study and organized the database. MM performed the statistical analysis. DM and MM wrote the first draft of the manuscript. All authors contributed to manuscript revision, read and approved the submitted version.

## Conflict of Interest Statement

The authors declare that the research was conducted in the absence of any commercial or financial relationships that could be construed as a potential conflict of interest.

## Supplementary Material

FIGURE S1 Example of research materials.
