# Supplementary figures and images for: Explicit Versus Implicit “Halal” Information: Influence of the Halal Label and the Country-of-Origin Information on Product Perceptions in Indonesia
Source: Front Psychol. 2018 Mar 22;9:382. doi: 10.3389/fpsyg.2018.00382 (PMC5874312; doi:10.3389/fpsyg.2018.00382)

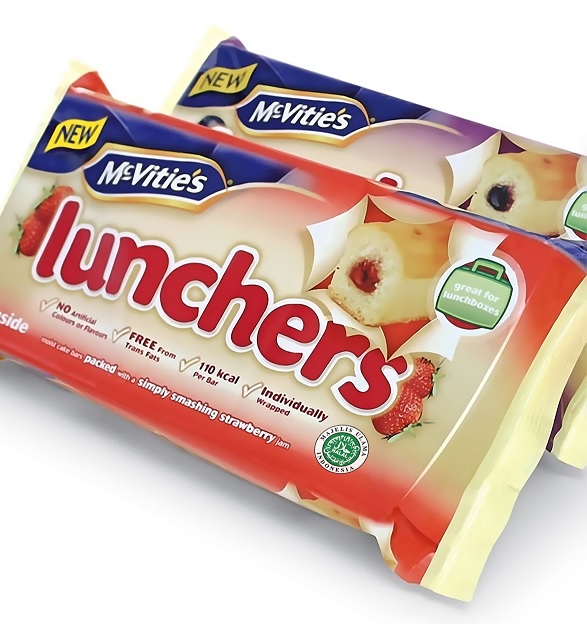

Supplement: FIGURE S1 — Example of research materials. [file Image_1.JPEG]
